# Adaptive PIF Control for Permanent Magnet Synchronous Motors Based on GPC

**DOI:** 10.3390/s130100175

**Published:** 2012-12-24

**Authors:** Shaowu Lu, Xiaoqi Tang, Bao Song

**Affiliations:** School of Mechanical Science and Technology, Huazhong University of Science and Technology, Wuhan 430074, China; E-Mails: guoting518@163.com (S.L.); xqtang@hust.edu.cn (X.T.)

**Keywords:** permanent magnet synchronous motor (PMSM), proportional integral feedforward (PIF) controller, speed reference, speed control system

## Abstract

To enhance the control performance of permanent magnet synchronous motors (PMSMs), a generalized predictive control (GPC)-based proportional integral feedforward (PIF) controller is proposed for the speed control system. In this new approach, firstly, based on the online identification of controlled model parameters, a simplified GPC law supplies the PIF controller with suitable control parameters according to the uncertainties in the operating conditions. Secondly, the speed reference curve for PMSMs is usually required to be continuous and continuously differentiable according to the general servo system design requirements, so the adaptation of the speed reference is discussed in details in this paper. Hence, the performance of the speed control system using a GPC-based PIF controller is improved for tracking some specified signals. The main motivation of this paper is the extension of GPC law to replace the traditional PI or PIF controllers in industrial applications. The efficacy and usefulness of the proposed controller are verified through experimental results.

## 1. Introduction

High-performance servo systems for permanent magnet synchronous motors (PMSMs) are essential in many applications such as precision engineering and industrial automation because of their advantages of high efficiency, high power factor and high power density [1–3]. As the control core of the speed control system for a PMSM, the servo drive generally uses a fixed proportional integral (PI) controller to complete the adjustment process. Although the PI controller has the advantages of simple algorithms and high reliability, the control performance is closely related to its structure and control parameters. Nevertheless, as a one-degree-of-freedom (1Dof) controller, even if a traditional PI controller is tuned to be optimal, it is difficult for the servo system to satisfy good load-disturbance rejection and good transient response simultaneously. Recently, controllers with two degrees of freedom (2Dof) are being used more and more frequently [4–6]. These controllers are aimed at responding swiftly to speed command changes and offering enough robustness against the uncertainties of the servo system, but more controller parameters must be tuned for the additional flexibility. Therefore, in order to meet the development requirements of high speed and high precision, it is thus desirable to have an intelligent 2Dof controller that can self-tune its control parameters according to the operating condition uncertainties.

Self-tuning strategies for industrial applications have been also widely investigated. Roughly speaking, there are two lines of thought to self-tune the controller parameters so that the controllers can adapt to the varying conditions: model-based methods and rule-based methods [7]. Fuzzy logic theory and neural network (NN) techniques were developed to provide rule-based methods that incorporate self-tuning capability to implement nonlinear algorithms [8–10]. They are characterized by a series of linguistic or weight statements in the controller, but there are obstacles to these methods. The learning cost of these methods is considerably large, and these control parameters cannot be adequately adjusted due to the nonlinear properties [11]. For the simplicity and feasibility of self-tuning strategies, model-based methods appear to be more suitable for adoption in real-time self-tuning situations. Alfaro *et al.* [7] proposed a robust tuning method for a 2Dof PI controller based on the model reference optimization procedure, but its effectiveness depended on the accuracy of the model, and the method might not sound solid when the estimated model conditions were violated in real applications.

In order to solve the problem considering the accuracy of the controlled model, many PID controllers based on a generalized predictive control (GPC) law have been proposed to improve the performance of the control system [12,13]. A GPC law is a model-based controller not requiring extra compensators, and has been successfully implemented in many industrial applications [14–17]. An important reason for the growing number of applications is the fact that its linear model can be obtained by identification techniques. Another important reason is its control performance deterioration caused by the identification errors of the controlled model can be prevented [18]. Therefore, by using GPC to tune 2Dof PI controller parameters online in this paper, not only can the stability of the speed control process be guaranteed, but control parameters can also be tuned automatically according to the changing characteristics of the controlled model.

In this paper, a PI plus feedforward (PIF) controller, in the form of a commercially available 2Dof PI controller, is used to improve the regulatory control performance and the closed-loop control system robustness simultaneously. The main motivation of this paper is to further advance the application of GPC in adaptive control for PMSM systems. There are possibly two contributions in design of a GPC-based PIF controller.

First, the mainly disadvantage of GPC law is that the computational expense to obtain the solution of an optimization process is considerable at each sampling time [19–21]. To solve the problem, a first-order controlled model is employed as the process object for the speed control system, and the parameters of this controlled model can be obtained directly by using a recursive least squares (RLS) algorithm with a low online computation burden. Moreover, Diophantine equations based on a first-order controlled model will be simplified obviously, so real-time performance of the GPC law can be guaranteed. Finally, the reference trajectory of a conventional GPC law was usually integrated as a smooth transition toward the desired trajectory or set point. However, in real industrial applications, this trajectory may not only cause a degradation of the closed-loop control performance, but increase the complexity of the GPC law for a PMSM system. In this paper, the trajectory will be ignored and a simplified cost function only requiring a future speed tracking error item and a penalization item on the torque current command is used.

Second, conventional GPC-based PID controllers use the future reference trajectory to obtain control performance as good as that of the GPC law, and have been designed to follow a step-type or a ramp-type reference command [18,22]. Frequent step-type speed commands for a PMSM will severely impact the electrical and mechanical agencies of the servo system, and a ramp-type command is discontinuous and just a special application, but the speed command for a commercial servo drive is usually required to be continuous and at least first-order differentiable. Meanwhile, due to the structural differences between a PIF controller and a PID controller, the coefficients of the GPC law need to be rearranged and keep in consonance with those of the PIF controller. In this paper, a GPC law can be straightforwardly replaced by a PIF controller, and the GPC-based PIF controller will be designed to follow many general reference curves for speed commands. However, it is noted that an arbitrary reference command does not necessarily satisfy the design requirements for the GPC-based PIF controller.

The paper is organized as follows: in Section 2, the system model of the PMSM is built in detail. In Section 3, the model parameters of a dynamic process object for a PMSM will be obtained in real time by using an RLS method, then, based on the controlled model and the future speed reference, a GPC law will supply a PIF controller with the suitable controller parameters to ensure good control performance. Experimental results are presented in Section 4 and conclusions are drawn in the final section.

## 2. System Model of PMSM

### 2.1. Speed Control System of PMSM

**Figure 1.**
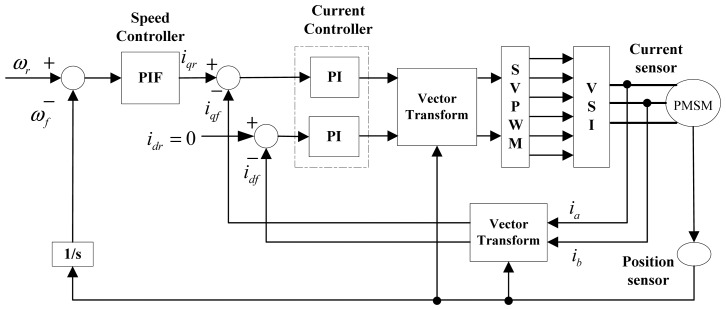
Vector-controlled PMSM servo system.

Under perfect field orientation and sensing technique conditions, the complicated coupled nonlinear dynamic performance of a PMSM can be significantly improved, whereby torque and flux can be tuned separately by two closed loops [23,24]. Thus accurate current feedback and position feedback are important factors in the realization of a high-precision and highly responsive closed control system [25]. A system configuration of a vector-controlled PMSM servo system is shown in Figure 1. In this vector control scheme, three-phase current is often detected by using a Hall current sensor, and position information is often obtained by using a high-resolution optical encoder.

**Figure 2.**
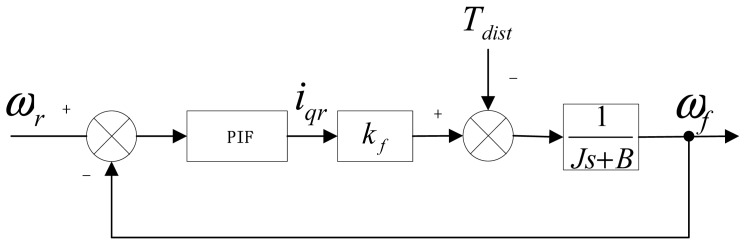
Average model of PMSM.

In the high-performance speed control system, a considerable high-bandwidth current loop is designed to ensure accurate current tracking and act as a current source amplifier within the current loop bandwidth [26]. Assuming the perfect current tracking and ignoring dead time in this paper, an average controlled model of a PMSM is shown in Figure 2. Accordingly, the mechanical dynamics can be reasonably described as:

(1) $$\omega_{f}\;(s)=\frac{k_{f}\;i_{\text{qr}}\;(s)-T_{\text{dist}}\;(s)}{J_{s}+B}$$

where *ω_f_* is feedback speed; *i_qr_* is expected torque current; *k_f_* is the torque coefficient related with flux linkage of PMSM; *J* is the system inertia including the load inertia; *T_dist_* is the external disturbances; *B* is the frictional torque coefficient.

The uncertainties of the servo system mainly include the load inertia and load torque. In the running of a servo system, system inertia may change. When the system inertia increases, the response of the servo system will speed up with system overshoot, which is likely to cause system instability. On the contrary, when the system inertia decreases, dynamic response will occur with system oscillation as well as turbulence. Meanwhile, the main role of the servo system is to drive the load operation, but in many industries, the load carried by servo system is not constant. Changes in the load torque will have a significant impact on servo control performance: in the running of a servo system, the sudden increase or reduce of load torque would result in fluctuations in servo speed control, affecting the accuracy of positioning and control performance.

Aside from the above reasons, the true controlled model may be more complex and of high-order since the current sensor and position sensor are nonlinear systems [27]. Actually, in order to better apply a GPC law to servo control, the effect of uncertainties is considered as the variation of model parameters. So the dynamic model of PMSM is rewritten as:

(2) $$\omega_{f}\;(s)=\frac{k_{f}\;i_{\text{qr}}\;(s)}{J_{s}+B}$$

### 2.2. Discrete-Time Model and Controller

Based on Equation (2), with the zero-order-hold (ZOH) conversion, the mathematical expression of controlled model can be described with the following discrete difference equation:

(3) $$\{\begin{array}{l} A(z^{-1})\omega_{f}\;(k)=B(z^{-1})i_{\text{qr}}\;\;(k-1)+\xi (k)/\Delta \\ A(z^{-1})=1-e^{-T_{s}B/J}z^{-1}=1+a_{1}z^{-1}\\ B(z^{-1})=k_{f}\;(1-e^{-T_{s}B/J})/B=b_{0} \end{array}$$

where *T_s_* is the sampling time of servo system; *a_1_* and *b_0_* are the estimated parameters; *ξ*(*k*) is white noise; Δ is the differential operator, Δ = 1 − *z*^−1^.

**Figure 3.**
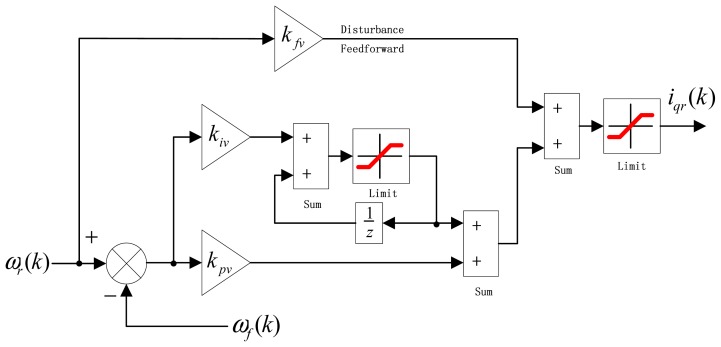
The structure of PIF controller.

The control structure of PIF controller is shown in Figure 3. In order to use GPC to tune the control parameters, the PIF controller can be described as:

(4) $$\Delta i_{\text{qr}}\;(k)=(k_{\text{pv}}\Delta +k_{\text{iv}}+k_{\text{fv}}\Delta )\omega_{r}\;(k)-(k_{\text{pv}}\Delta +k_{\text{iv}})\omega_{f}\;(k)$$

(5) $$i_{\text{qr}}\;(k)=i_{\text{qr}}\;\;(k-1)+\Delta i_{\text{qr}}\;(k)$$

where *k_pv_* is velocity loop proportional gain; *k_iv_* is velocity loop integral gain; *k_fv_* is velocity loop feedforward gain. *i_qr_* usually need to be limited, and Δ*i_qr_* is used for GPC to replace PIF controller.

## 3. The design of Adaptive PIF Controller

**Figure 4.**
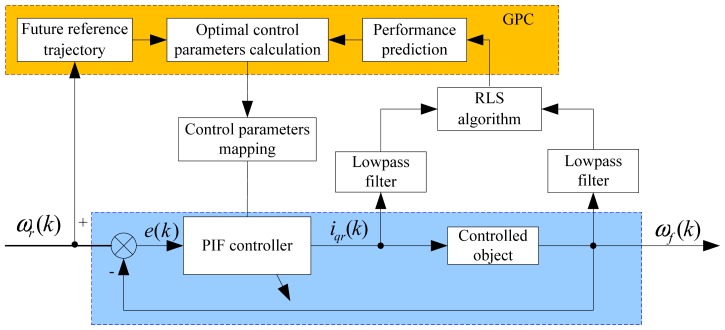
The principle diagram of a PIF control-based GPC.

The basic function of the GPC law is to calculate a sequence of future control signals in such a way that it minimizes a cost function defined over a prediction horizon [28,29]. In this paper, the GPC-based PIF controller mainly includes performance prediction, future reference trajectory and control parameters calculation. The structure of a GPC-based PIF controller is presented in Figure 4. Firstly, torque current and actual speed are sampled for obtaining a controlled model in every control period, and a recursive least squares (RLS) algorithm is used to update the model parameters. Then, based on the controlled model and future reference trajectory, the predicted output can be obtained and the calculation of future control values can be achieved. Finally, future control values will supply the PIF controller with suitable control parameters online.

### 3.1. The Identification of Model Parameters

In order to obtain predictions of the speed control performance, continuous recognition should be applied to the model parameters of the controlled object. The model parameters can be generally obtained by a RLS algorithm, so the parameters in the polynomial of *A*(*z*^−1^) and *B*(*z*^−1^) in Equation (3) are calculated using the following recursive least squares method [30]:

(6) $$\hat{\theta }(k)=\hat{\theta }(k-1)+K(k)[\omega_{f}\;(k)-\hat{\theta }(k-1)\varphi (k-1)]$$

(7) $$K(k)=\frac{X\;(k-1)\varphi \;(k-1)}{\alpha^{*}+\varphi^{T}\;(k-1)X\;(k-1)\varphi \;(k-1)}$$

(8) $$X(k)=\frac{1}{\alpha^{*}}\left[X\;(k-1)-\frac{X\;(k-1)\varphi^{T}\;(k-1)X\;(k-1)\varphi \;(k-1)}{\alpha *+\varphi^{T}\;(k-1)X\;(k-1)\varphi \;(k-1)}\right]$$

(9) $$\{\begin{array}{l} \hat{\theta }(k)=[{\hat{a}}_{1}(k),{\hat{b}}_{0}(k)]\\ \varphi \;(k-1)=[-\omega_{f}\;(k-1),i_{\text{qr}}\;(k-1)] \end{array}$$

where α* is the forgetting factor; X(0) = *α*_Γ_*I* (0 < *α*_Γ_ < ∞).

### 3.2. Performance Prediction

To simplify the GPC law, the trajectory will be ignored, and a cost function of the speed control system is designed by using output error and quadratic indicators form of weighted control increments:

(10) $$J=\sum_{j=N_{1}}^{N_{2}}{\left[\omega_{f}\;(k+j)-\omega_{r}\;(k+j)\right]}^{2}+\lambda \sum_{j=1}^{N_{u}}\Delta i_{\text{qr}}{\left(k+j-1\right)}^{2}$$

where *N*_1_ is minimum predictive horizon, usually choose *N*_1_ = 1; *N*_2_ is maximum predictive horizon; *N_u_* is control horizon, generally *N_u_* ≤ *N*_2_; *λ* is the control increment weighting factor.

Because *B*(*z*^−1^) is zero order polynomial, simplified Diophantine Equations (11) and (12) are solved to obtain *ω_f_* (*k + j*):

(11) $$1=E_{j}(z^{-1})A(z^{-1})\Delta +z^{-j}F_{j}(z^{-1})$$

(12) $$E_{j}(z^{-1})B(z^{-1})=G_{j}(z^{-1})$$

(13) $$\{\begin{array}{l} E_{j}(z^{-1})=e_{0}+e_{1}z^{-1}+\cdots +e_{j}z^{-j+1}\\ F_{j}(z^{-1})=f_{0}^{j}+f_{1}^{j}z^{-1}\\ G_{j}(z^{-1})=g_{0}+g_{1}z^{-1}+\cdots +g_{j}z^{-j+1} \end{array}$$

Then the *j* steps ahead predictive output is obtained by following equation:

(14) $$\omega_{f}\;(k+j)=G_{j}\Delta i_{\text{qr}}\;(k+j-1)+F_{j}\omega_{f}\;(k)+E_{j}\xi \;(k+j)$$

Obviously, in the right hand part of Equation (14), the first two terms and the third are irrelevant. If the first two are regarded as the optimal prediction, then the third is the prediction error, that is, the optimal prediction controlled object can be expressed as:

(15) $${\hat{\omega }}_{f}\;(k+j)=G_{j}\;(z^{-1})\Delta i_{\text{qr}}\;(k+j-1)+F_{j}\;(z^{-1})+\omega_{f}\;(k)$$

The vector form of Equation (14) is expressed as:

(16) $$\omega_{f}={\mathrm{Gi}}_{\text{qr}}+{F\omega }_{f}\;(k)+E$$

(17) $${\omega_{f}}^{T}=[\omega_{f}\;(k+1),\cdots ,\omega_{f}\;(k+N_{2})]$$

(18) $${i_{\text{qr}}}^{T}=[\Delta i_{\text{qr}}(k),\cdots ,\Delta i_{\text{qr}}(k+N_{u}-1)]$$

(19) $$F^{T}=[F_{1}(z^{-1}),\cdots ,F_{N_{2}}(z^{-1})]$$

(20) $$E^{T}=[E_{1}\xi (k+1),\cdots ,E_{N_{2}}\xi (k+N_{2})]$$

(21) $$G=\left[\begin{array}{cccc} g_{0} & & & \\ g_{1} & g_{0} & & \\ \vdots & & & \\ g_{N_{u}-1} & g_{N_{u}-1} & \cdots & g_{0}\\ \vdots & & & \\ g_{N_{2}-1} & g_{N_{2}-1} & \cdots & g_{N_{2}-N_{u}} \end{array}\right]$$

The vector form of the cost Function in Equation (10) can be obtained:

(22) $$J={\left(\omega_{f}-\omega \right)}^{T}(\omega_{f}-\omega )+\lambda {i_{\text{qr}}}^{T}i_{\text{qr}}$$

(23) $$\omega^{T}=[\omega_{r}\;(k+1),\cdots ,\omega_{r}\;(k+N_{2})]$$

Since Equation (22) is quadratic in *i_qr_*, the optimal solution with respect to *i_qr_* is obtained by:

(24) $$i_{\text{qr}}={\left(G^{T}G+\lambda i\right)}^{-1}G^{T}[\omega -{F\omega }_{f}]$$

The use of the first element of *i_qr_* gives the following control law:

(25) $$\Delta i_{\text{qr}}(k)=P(z^{-1})\omega_{r}\;(k+N_{2})-F(z^{-1})\omega_{f}\;(k)$$

(26) $$\{\begin{array}{l} {}[p_{N_{1}}\cdots p_{N_{2}}]=[10\cdots 0]{\left(G^{T}G+\lambda i\right)}^{-1}G^{T}\\ P(z^{-1})=p_{N_{2}}+p_{N_{2}-1}z^{-1}+\cdots +p_{N_{1}}z^{-(N_{2}-N_{1})}\\ F(z^{-1})=p_{N_{1}}F_{N_{1}}(z^{-1})+p_{N_{1}+1}F_{N_{1}+1}(z^{-1})+\cdots +p_{N_{2}}F_{N_{2}}(z^{-1})=f_{0}+f_{1}z^{-1} \end{array}$$

### 3.3. Future Reference Trajectory

#### Remark 1

Compared with the increment control output in [18,22], Equation (25) is more simple, only requiring a future speed command item and a feedback speed item. However, the future speed command item cannot be straightforwardly achieved in the PIF controller. To apply the GPC law to the PIF controller, assuming the speed command curve for PMSM is arbitrary-order continuously differentiable at some points, the future reference trajectory can be expressed by the following equation by using Taylor series expansion:

(27) $$\omega_{r}\;(k+j)=\omega_{r}\;(k)+g(k,j)\Delta \omega_{r}\;(k)$$

where *g*(*k*, *j*) is related to *k* and *j*. Detailed expressions of Equation (27) are given in Appendix A.

#### Remark 2

If the speed command cannot be expressed as Equation (27), the polynomial of GPC will not match with the polynomial of the PIF controller, and the GPC law will not supply the PIF controller with the suitable control parameters. When a PMSM runs at constant velocity, the servo drive is required to set the rise time and the drop time. Therefore, the rise stage and the drop stage of speed command are usually set as a continuously differentiable curve, e.g., trapezium-type or exponent-type curve, in this case, *g*(*j*) is only related to *j* and Equation (27) can be simplified as:

(28) $$\omega_{r}\;(k+j)=\omega_{r}\;(k)+g(j)\Delta \omega_{r}\;(k)$$

### 3.4. Optimal Control Parameters Calculating

Using Equation (28), the first term on the right hand side of Equation (25) is rewritten as:

(29) $$\{\begin{array}{l} P\;(z^{-1})\omega_{r}\;(k+N_{2})=p_{r}\omega_{r}\;(k)+p_{s}\Delta \omega_{r}\;(k)\\ p_{r}=\sum_{j=N_{1}}^{N_{2}}p_{j}\\ p_{s}=\sum_{j=N_{1}}^{N_{2}}p_{j}\;g\;(j) \end{array}$$

Using Equation (29), the GPC law Equation (25) is rewritten as:

(30) $$\Delta i_{\text{qr}}(k)=(p_{r}+p_{s}\Delta )\omega_{r}\;(k)-(f_{0}+f_{1}-f_{1}\Delta )\omega_{f}\;(k)$$

Based on $f_{0}^{j}+f_{1}^{j}=1$, so the following equations can be obtained:

(31) $$\{\begin{array}{l} f_{0}+f_{1}=\sum_{j=N_{1}}^{N_{2}}p_{j}\\ p_{r}=f_{0}+f_{1} \end{array}$$

Then comparing Equation (30) with Equation (4), the PIF controller gain parameters are calculated by the GPC law:

(32) $$\{\begin{array}{l} k_{\text{pv}}=-f_{1}\\ k_{\text{iv}}=f_{0}+f_{1}\\ k_{\text{fv}}=p_{s}+f_{1} \end{array}$$

With the above discussion, the implementation of a GPC-based PIF controller design is summarized as follows:

Step 1: Given the related parameters for an RLS law and a GPC algorithm.
Step 2: The controlled model parameters *a*_1_ and *b*_0_ are updated online by the RLS law.
Step 3: Based on the controlled model, Diophantine Equations (11) and (12) are solved to obtain *ω_f_* (*k + j*) and Δ*i_qr_*(*k*) is obtained by Equation (25).
Step 4: Speed command can be expressed as Equation (28), then, the PIF controller gain parameters can be tuned by Equation (32).
Step 5: Set *k* = *k* + 1 and go to step 2.

## 4. Experiment

**Figure 5.**
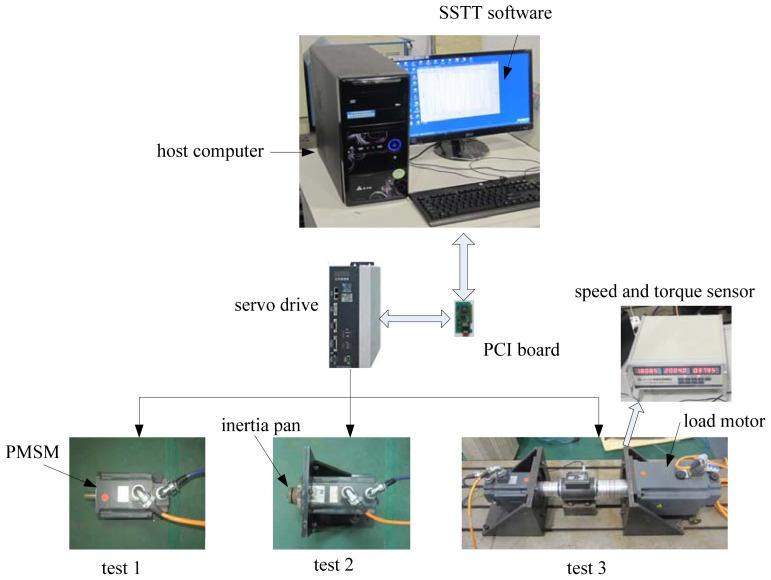
The apparatus for the experimental platform.

The apparatus for the experimental platform contains three major parts and some data transferring buses, as shown in Figure 5. These three major parts are: (1) PC-based PCI motion controller with sampling time equal to 5 ms; (2) AC servo drive with position, velocity and torque control modes using a DSP plus a FPGA, where DSP TMS320F2812 mainly accomplishes in real-time control algorithms during motion control, and FPGA EP2C8Q208C8N is responsible for the analysis and realization of absolute ruler and NCUC-Bus protocols; (3) PMSM with the parameters described in Table 1, increment encoder with 2,500 pulse/revolution is fixed in the PMSM. Through the PCI controller, the PC sends the speed command and control parameters to the servo drive, receives expected torque current and feedback velocity from the servo drive for the identification of model parameters, and implements the RLS algorithm and proposed GPC-based PIF design every 5 ms by a commercial Servo Self-adapted Turning Tool (SSTT) software based on the VC environment.

In the experimental tests, the maximum value of the speed command is set to 1,200 r/min, its rise time and drop time are set to 1 s. Finally, all experimental data from the SSTT are plotted by using Matlab 6.5. The related parameters for the RLS algorithm and the GPC law are: *N*_1_ = 1, *N*_2_ = 10, *N_u_* = 2, *λ* = 0.01, and α* = 0.94. To evaluate the control performance and confirm the effectiveness of the proposed strategy, three different tests, which all consider a traditional PI controller and a traditional PIF controller, have been taken on the experiment platform.

### 4.1. Test 1 without Applied Load

**Figure 6.**
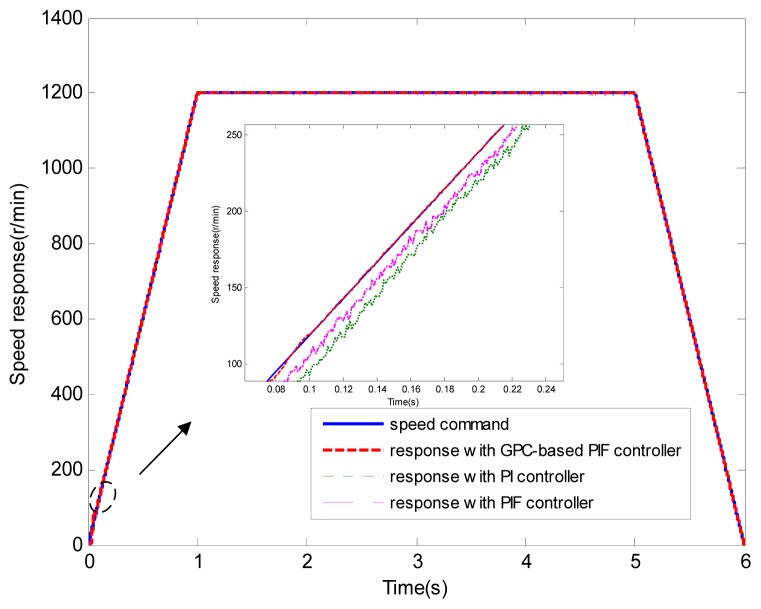
Speed response (test 1).

**Figure 7.**
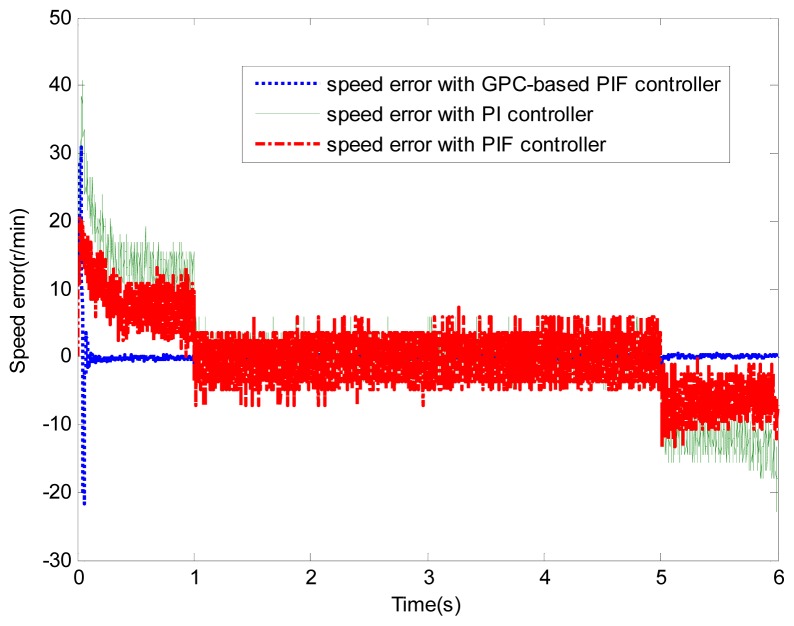
Speed error (test 1).

**Figure 8.**
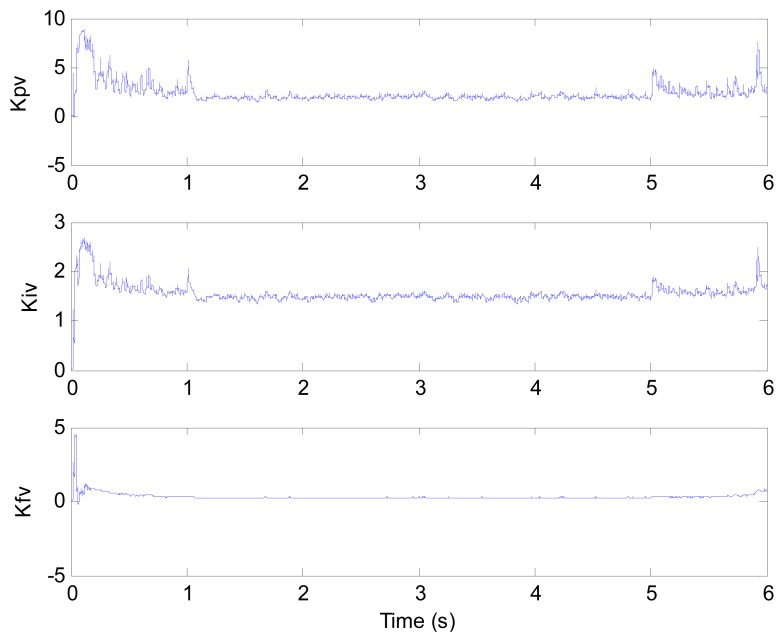
Control parameters (test 1).

**Figure 9.**
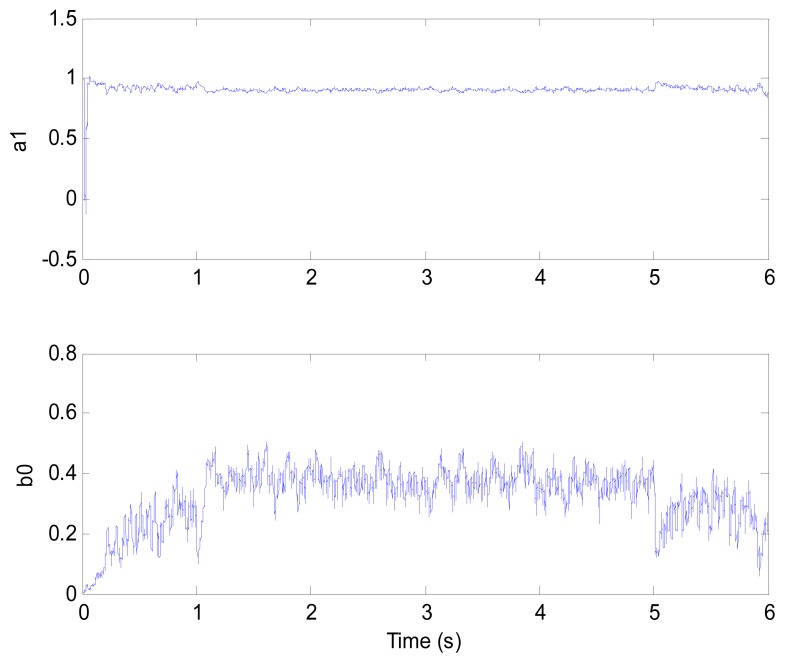
Model parameters (test 1).

Figures 6 and 7 show the speed response and speed error for the traditional PI controller, traditional PIF controller and GPC-based PIF controller. It is clear that traditional PI controller and traditional PIF controller do not meet the precision control requirement, and a large speed error exists in the whole control process, especially in the rise stage and the drop stage. Meanwhile, the performance of the speed response based on the adaptive PIF controller is obviously improved in the whole process, except in the initial stage, because model parameters are not completely convergent. PIF controller gains are shown in Figure 8, because more torque is required in the rise stage and the drop stage, controller gains of the rise stage and the drop stage is larger than those of the steady stage. The identification result for model parameters using the RLS algorithm is shown in Figure 9, it can be seen that model parameters in uniform velocity stage are relatively stable without applied load.

### 4.2. Test 2 with Applied Load Inertia

**Figure 10.**
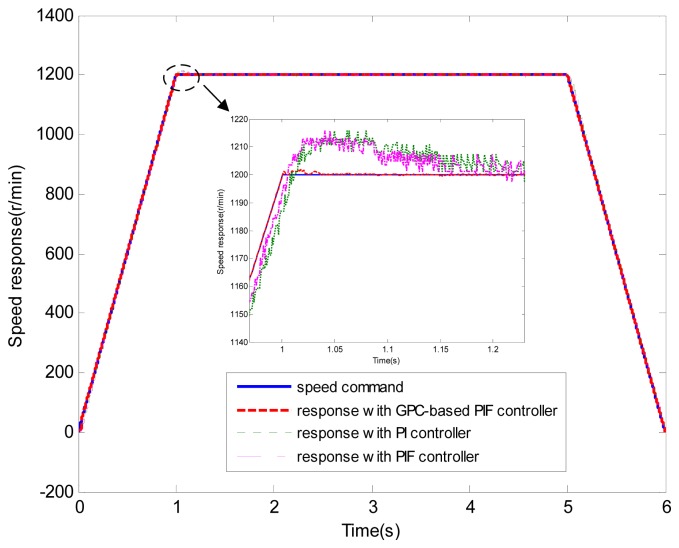
Speed response (test 2).

**Figure 11.**
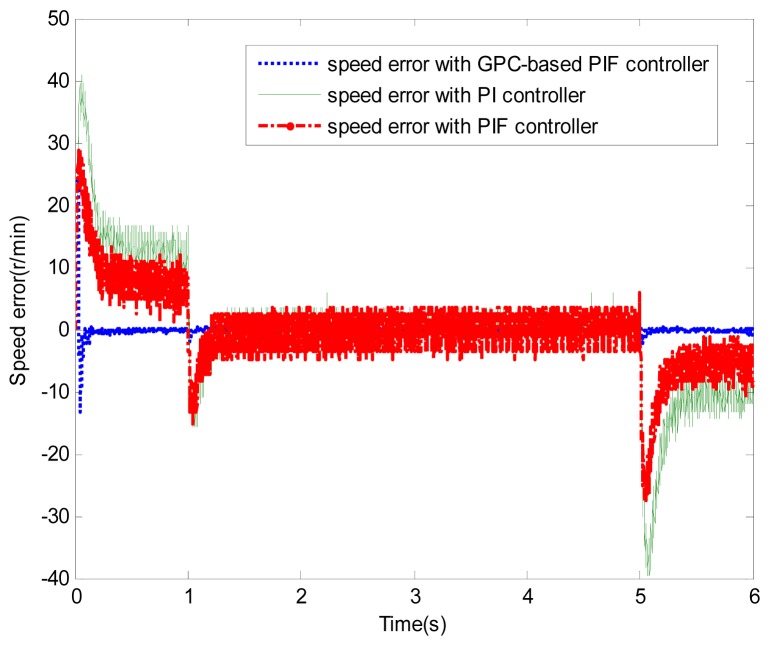
Speed error (test 2).

**Figure 12.**
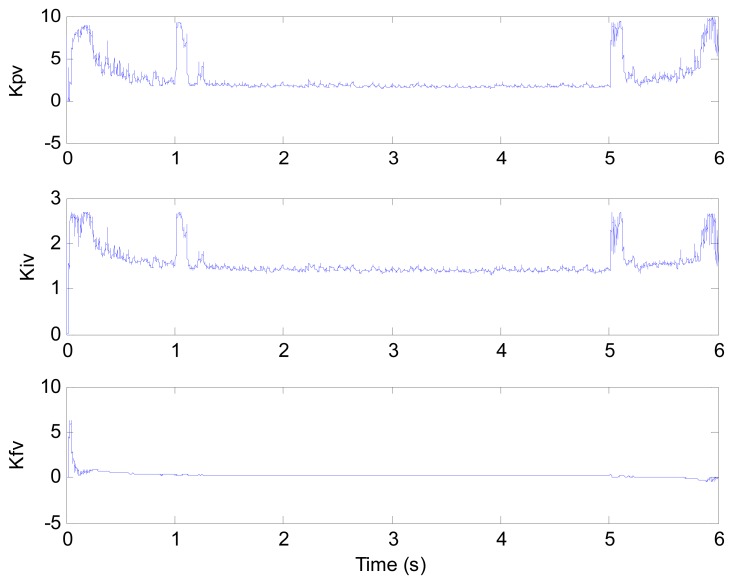
Control parameters (test 2).

**Figure 13.**
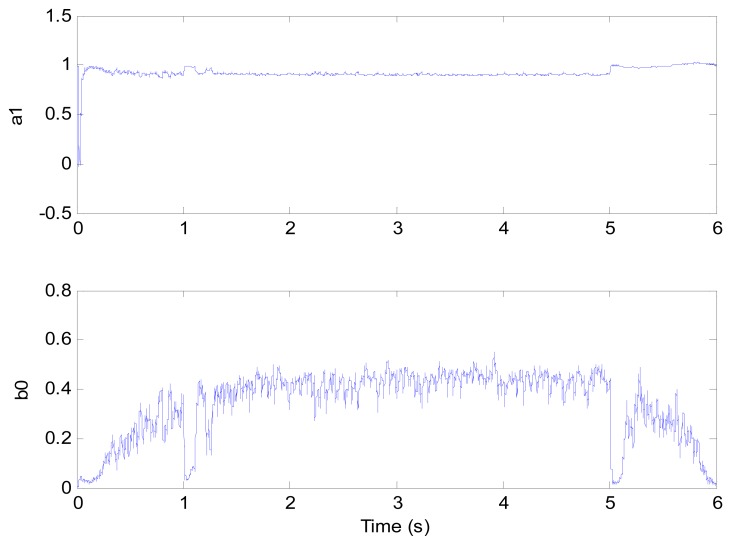
Model parameters (test 2).

In this test, the load inertia is a triple rotor inertia, and is applied to confirm the robustness and the effectiveness of the proposed controller. It is noted that the control gains of traditional PI controller and traditional PIF controller are the same as in test 1. Figures 10 and 11 show the speed response and speed error for a traditional PI controller, traditional PIF controller and GPC-based PIF controller. It is clear that the traditional PI controller and traditional PIF controller both lead to large speed errors and system overshoot in the control process. Meanwhile, the adaptive PIF controller can continue to maintain fairly good control performance in the whole control process, but the initial model mismatch still exists. GPC-based PIF controller gains and model parameters are shown in Figures 12 and 13, because system inertia becomes larger and leads to speed overshoot in the transition point between the rise stage and the steady stage, controller gains of the transition stage are required to become larger to overcome the larger inertia.

### 4.3. Test 3 with Applied Load Torque

**Figure 14.**
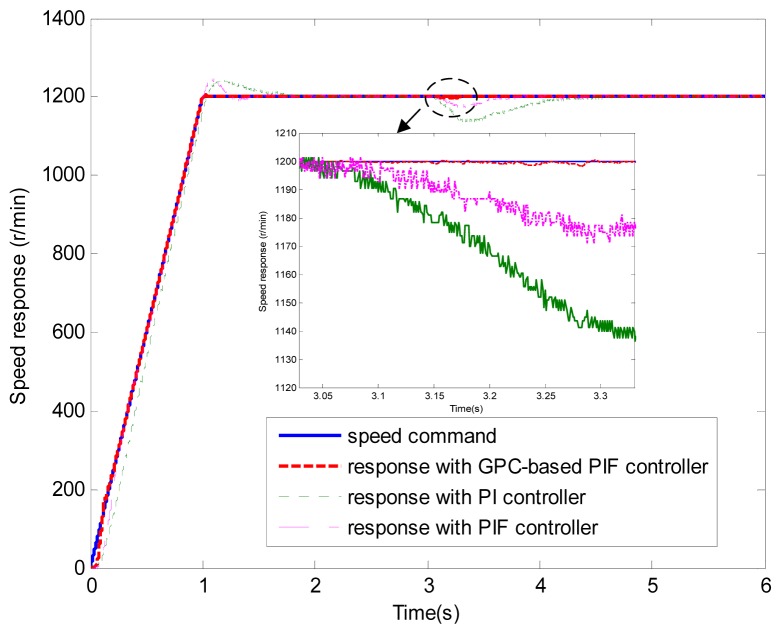
Speed response (test 3).

**Figure 15.**
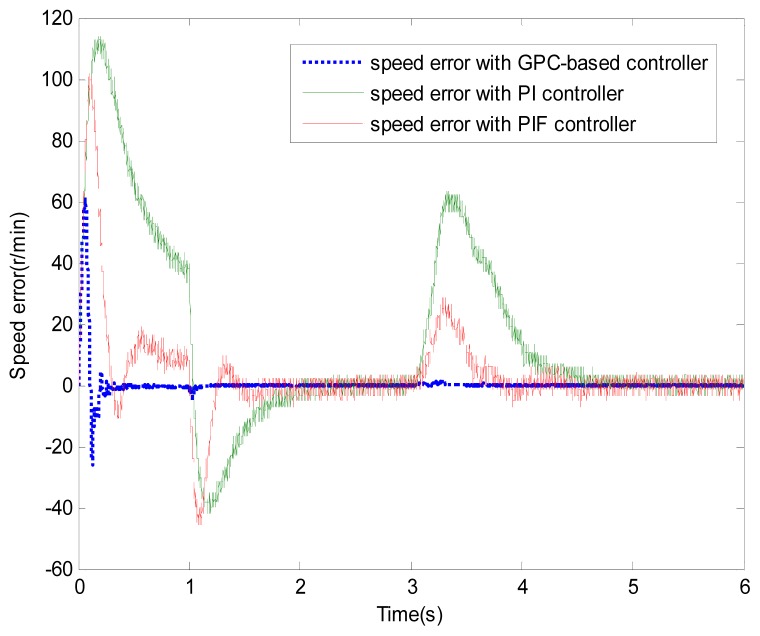
Speed error (test 3).

**Figure 16.**
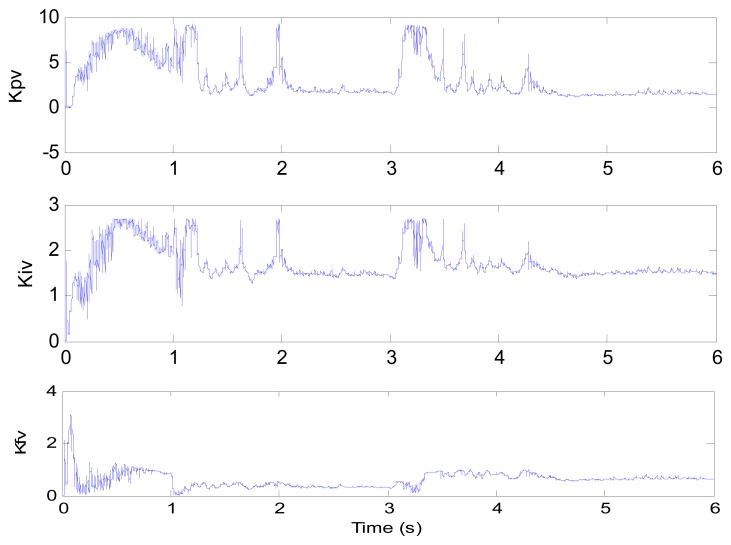
Control parameters (test 3).

**Figure 17.**
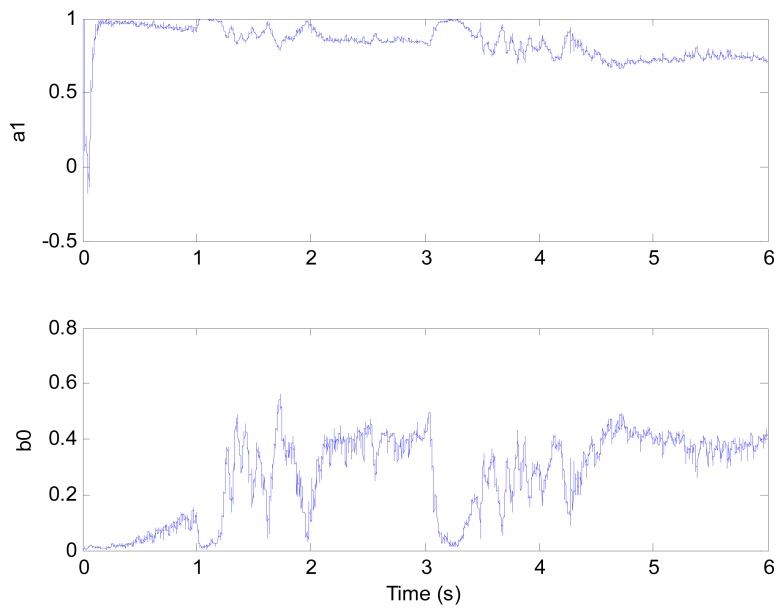
Model parameters (test 3).

In this test, load torque is applied to confirm the robustness and the effectiveness of the proposed controller. The initial load torque is set to 2 Nm, and became to be 9 Nm when *t* = 3 s. It is noted that the control gains of the traditional PI controller and traditional PIF controller are still the same as in test 1. Figures 14 and 15 show the speed response and speed error for the three controllers. When 0 < *t* ≤ 3 s, it is clear that traditional PI controller and traditional PIF controller both lead to larger speed errors and system overshoot than those in test 2. When *t* ≥ 3 s, because of the variation of load torque, the actual speed drops and begins to keep speed tracking after 1 s. Meanwhile, the adaptive PIF controller can continue to maintain fairly good control performance in the whole control process especially when *t* ≥ 3 s. The initial model mismatch still exists, but the control performance is superior for the adaptive PIF controller. PIF controller gains and model parameters are shown in Figures 16 and 17, because load torque becomes larger and makes servo speed drops when *t* ≥ 3 s, controller gains of the transition stage is required to become larger to overcome the load torque.

## 5. Conclusions

The speed control systems of the PMSMs employed in various industries are almost always controlled by traditional PI and PIF controllers. In order to meet the development requirements of high performance servo systems, this paper has proposed a new GPC-based PIF controller to increase the robustness of servo systems. In this method, controlled model parameters can be obtained online by an RLS algorithm. Based on the controlled model, a high performance and simplified GPC supplies the PIF controller with suitable control parameters according to the uncertainties in the operating conditions. The experimental results show a good control performance and strong robustness of the speed control system can be maintained.

## Figures and Tables

**Table 1.** Specification of the PMSM.

**Name**	**Values**
Phase current	3.7 A
The number of poles	3
Rated torque	4.5 Nm
Rotor inertia	0.00067 kgm^2^
Rated speed	1,200 r/min

## Appendix A

Assuming the speed command curve for a PMSM is arbitrary-order continuously differentiable at *k* = *0*, Taylor series expansion can be expressed as following equation:

(A.1) $$\omega_{r}\;(k)=\sum_{n=0}^{\infty }\frac{\omega_{r}^{\left(n\right)}(0)}{n!}k^{n}$$

So, Taylor series expansions of *ω_r_*(*k + j*) and Δ*ω_r_*(*k*) can be obtained:

(A.2) $$\omega_{r}\;(k+j)=\sum_{n=0}^{\infty }\frac{\omega_{r}^{\left(n\right)}(0)}{n!}{\left(k+j\right)}^{n}$$

(A.3) $$\Delta \omega_{r}\;(k)=\omega_{r}\;(k)-\omega_{r}\;(k-1)=\sum_{n=0}^{\infty }\frac{\omega_{r}^{\left(n\right)}(0)}{n!}[k^{n}-{\;(k-1)}^{n}]$$

Substituting Equations (A.1), (A.2) and (A.3) into Equation (27), *g*(*k*, *j*) can be obtained:

(A.4) $$g(k,j)=\frac{\sum_{n=0}^{\infty }\frac{\omega_{r}^{\left(n\right)}(0)}{n!}[{\left(k+j\right)}^{n}-k^{n}]}{\sum_{n=0}^{\infty }\frac{\omega_{r}^{\left(n\right)}(0)}{n!}[k^{n}-{\;(k-1)}^{n}]}$$

For example, a three-order polynomial function is chosen as the rise stage curve for speed command and can be expressed as following equation:

(A.5) $$\omega_{r}\;(k)=ck^{3}$$

where, *c* is a constant. So, substituting Equation (A.5) into Equation (A.4), the corresponding *g*(*k*, *j*) can be obtained:

(A.6) $$g(k,j)=\frac{{\left(k+j\right)}^{3}-k^{3}}{k^{3}-{\;(k-1)}^{3}}$$
